# Sublingual immunotherapy adherence in patients with allergic rhinitis: Effects of an intervention based on the information-motivation-behavioral skills model

**DOI:** 10.1016/j.heliyon.2023.e22929

**Published:** 2023-11-28

**Authors:** Qian Wang, Ruifang Zhu, Yingzi Cao, Yan Ning, Yaoqing Feng, Yan Feng, Shifan Han

**Affiliations:** a School of Nursing, Shanxi Medical University, Taiyuan, China; b First Hospital of Shanxi Medical University, Taiyuan, China

**Keywords:** Information-motivation-behavioral skills model, Allergic rhinitis, Sublingual immunotherapy, Adherence

## Abstract

**Objective:**

This study aimed to confirm the efficacy of an intervention based on the information-motivation-behavior skills (IMB) model in improving the sublingual immunotherapy adherence score, medication beliefs score, self-efficacy score, and medication knowledge score of patients with allergic rhinitis.

**Methods:**

A total of 120 patients with allergic rhinitis from an otorhinolaryngology clinic were divided into the control group (n = 60) and experimental group (n = 60); the study was conducted from August 2021 to March 2022. The control group received routine intervention, whereas the experimental group received intervention based on the IMB model. The medication adherence, medication beliefs, self-efficacy, and medication knowledge levels of the two groups were evaluated at baseline, post-intervention, at 1-month follow-up, and at 3-month follow-up.

**Results:**

After intervention, a total of 116 patients completed the trial in the experimental and control groups (58 cases each). The results revealed differences in the scores of medication adherence, belief, self-efficacy, and knowledge between the two groups post-intervention, at 1-month follow-up, and at 3-month follow-up (P < 0.05). Further, the scores of the four indexes of the two groups were different with time, and better changes were noted among experimental group participants.

**Conclusion:**

Implementing interventions based on the IMB model for people receiving sublingual immunotherapy for allergic rhinitis can effectively improve patients' medication beliefs, self-efficacy, and knowledge of medication, thereby improving their medication adherence, ensuring efficacy, and providing medication care for outpatients.

## 1 Introduction

Allergic rhinitis (AR) is one of the most common diseases encountered in otolaryngology head and neck surgery; AR is an inflammatory disease of the nasal mucosa mediated by immunoglobulin E after exposure to allergens [1,2]. The typical symptoms include itchy nose, nasal congestion, clear runny nose, sneezing, allergic conjunctivitis, asthma, otitis media, nasal polyps, and other complications that may occur if treatment is not timely or appropriate. It greatly affects people's quality of life and imposes a huge financial and psychological burden on patients [3].

The main treatments for AR are symptomatic drug therapy and allergen specific immunotherapy (ASIT). Currently, the first line of pharmacological treatment includes antihistamines, leukotriene receptor blockers, and nasal hormone sprays, but these drugs cannot be used for long periods of time and are prone to tolerance and even side effects. To date, however, ASIT is the only treatment that addresses the etiology of AR [4,5]. The two main routes of administration are subcutaneous immunotherapy (SCIT) and sublingual immunotherapy (SLIT) [6]. Compared to SCIT, SLIT is safer and can be self-administered without medical supervision, making it the preferred route of desensitization [7]. Recent research has reported that SLIT adherence is generally poor and needs to be improved [8]. Current interventions for adherence mostly use interventions common to chronic diseases, such as health education and telephone follow-up, to improve patients' medication adherence [9]; however, they neglect patients' motivation and self-efficacy, which are the main factors affecting patients' medication adherence [10].

The information-motivation-behavioral skills (IMB) model uses the three dimensions of information, motivation, and behavioral skills to explain the complex process of behavioral change in human beings, and it includes several variables that influence medication adherence [11]. In recent years, interventions based on the IMB model have been widely applied to support medication adherence in patients with diabetes, asthma, HIV, and other diseases, and have achieved satisfactory results [[12], [13], [14]]. The aim of this study was to validate the effectiveness of an IMB model-based intervention program on SLIT adherence in AR patients, and to provide new ideas for clinical medication care for AR patients.

## 2 Method

### 2.1 Study design

This study used the historical controls (HCT) method and employed a non-randomized controlled trial design (Chinese clinical trial registration number: ChiCTR2100046723). Using the HCT method, patients who met the inclusion criteria before December 1, 2021 were included in the control group and those who met the inclusion criteria after December 1, 2021 were included in the experimental group.

### 2.2 Participants

The participant inclusion criteria were as follows: patients who met the diagnostic criteria of the Chinese guideline for diagnosis and treatment of allergic rhinitis (2022, revision) [2] and were diagnosed with AR; patients whose skin prick test results were positive for antigens, for whom dust mites were the main allergens, and who reported taking no antihistamines within 1 week; patients who received SLIT and the drug used was Changdi Dust Mite Drops (Zhejiang Iwu Biotechnology Co., Ltd.); patients aged ≥18 years; and patients who provided informed consent, voluntarily participated in this study, and cooperated voluntarily.

Patients with a previous history of mental illness and cognitive impairment and those with comorbid chronic conditions such as eczema, asthma, diabetes, and hypertension were excluded.

### 2.3 Sample size estimation

The sample size required for this study was calculated according to the formula of comparing the means of the two samples, which is as follows:

$$N_{1}=N_{2}=2{\left[\sigma \left(t_{\alpha /2}+t_{\beta }\right)/\left(\mu_{1}-\mu_{2}\right)\right]}^{2}$$

where Uα and Uβ are the U values corresponding to α and β. The test level α was taken as 0.05 (bilateral) and β as 0.1. Referring to Mann's [15] study, medication adherence was calculated as an indicator, yielding μ1-μ2 = 2.43 and σ = 3.65. Substituting the values into the formula resulted in N1=N2=48 cases; considering the 20 % missed follow-up rate, we derived a sample of 60 cases in each group, totaling 120 cases.

### 2.4 Study intervention

The IMB model-based intervention program was developed and implemented in the experimental group.

(1) Subject group: The subject group based on the IMB model intervention program was established, including two experts in the treatment of allergic reactions in otorhinolaryngology head and neck surgery, two master's degree students in nursing, one nurse practitioner in otorhinolaryngology, and one professor of psychology, to provide knowledge and information to the participants.
(2) Intervention program: The interventions were developed by considering the three dimensions of information, motivation, and behavioral skills. We used the WeChat platform (WeChat public platform and WeChat group chat) to collect relevant information from patients, conduct follow-up management, and evaluate the effects of the intervention program on the level of medication adherence, medication beliefs, self-efficacy, and medication knowledge of AR patients who received SLIT. The following steps were taken for each dimension. ① Information intervention: We referred to *Chinese guideline for diagnosis and treatment of allergic rhinitis* [2] and *Chinese guideline on sublingual immunotherapy for allergic rhinitis and asthma* [16] to compile a promotional manual to be reviewed by two ear, nose, and throat (ENT) specialists in allergology treatment and one ENT nurse-in-charge; we also used the WeChat platform to share popular science articles and videos with patients. ② Motivational intervention: We referred to Zhang's [17] motivational interview outline on improving medication adherence in asthma patients to develop the interview outline for the three motivational interviews conducted in the current study, which was reviewed by a professor of psychology. ③ Behavioral skills intervention: We made medication record cards and daily punch cards on the WeChat public website. The detailed steps are shown in Table 1.

**Table 1:** Intervention program based on the IMB model.

Information	Motivation	Behavioral skills	Intervention steps
① Educational booklet: contains information on treatment of AR, importance of desensitization, principles of SLIT, administration of SLIT, observation of efficacy, precautions, management of adverse reactions, how to resume medication after missed doses, etc.	First time (at study inclusion): ● Assessing patients' previous medication use, medication adherence, and motivation to use medication. ● Instructing patients to choose a score (0−10) on their willingness to comply with their medication to make them aware of the conflict between their own willingness to take medication and their medication behavior, to balance the pros and cons, and to motivate them to change. ● Asking patients their reason(s) for choosing their particular motivation score. Estimated length of intervention: 20−30 min	Medication administration record cards: electronic versions of the cards are produced according to the medication administration schedule and are available for patients to print out and use.	Subject: ● Establishing a WeChat platform. ● Recruiting patients under license. ● Ongoing from hospital admission to 3 months post-treatment.
② Providing knowledge and answering patients' queries on a daily basis on the WeChat group: one ENT specialists in allergology, one ENT nurse, and two postgraduate nursing students provide information and answer questions.	Second (first month into the study): ● Guiding patients based on scores on their willingness to comply with their medication. ● Based on the patient's score, an interview is conducted using the appropriate interview topic: ➢ Score 1−5: the interview topic is “Benefits and disadvantages of medication use: current situation and expectations" ➢ Score 6−7: the interview topic is “Asking about medication concerns, responding to concerns, and motivation" ➢ Score >7: the interview theme is “Encouragement and making suggestions" Estimated length of intervention: 20−30 min	Daily medication reminders on WeChat: including names and dosages. Frequency: Once a day	Health care professionals: ● Providing daily information on AR desensitization treatment (5 p.m.−7 p.m.). ● Assessing and answering questions submitted by patients as soon as possible.
③ WeChat public website for sharing articles: knowledge about the disease, knowledge about SLIT, precautions, etc.	Third (third month of inclusion in the study)： ● The method is the same as the second interview, reinforcing the content of the previous two interviews and reviewing and summarizing previous medication use. ● Asking patients about their previous medication experience, summarizing any problems, and providing advice and guidance. Estimated length of intervention: 20−30 min	WeChat public daily punch card: WeChat automatically sends a message reminder at 8 a.m. every day, and the researcher completes the daily verification.	Patients： ● Asking an existing question. ● Sharing about treatment experience.

AR: allergic rhinitis; SLIT: sublingual immunotherapy; ENT: ear, nose, and throat.

### 2.5 Usual care

The control group received routine medication instruction, including verbal health education by doctors and nurses, and received the medication knowledge booklet provided by the manufacturer of SLIT. Further, other support measures such as patiently answering questions raised by the patients, establishing contact with patients, and regular telephone follow-up and outpatient follow-up to answer patients' questions were undertaken.

### 2.6 Measurements

The Medication Adherence Scale developed by Dong [18] was used in this study. The scale consists of two dimensions—reasons for the patient's non-adherence and complexity of the medication regimen—with a total of seven entries. A 5-point scale is used, with “always,” “often,” “sometimes,” “rarely,” and “never” scored on a scale of 1–5. Scores range from 7 to 35; higher scores signify better medication adherence. The total Cronbach alpha of the scale ranged from 0.701 to 0.827, with a retest reliability of 0.815.

The Chinese modified version of the Beliefs About Medication Scale (BQM) consists of two dimensions, medication necessity and medication apprehension, with 10 entries and a score range of 10–50 on a 5-point scale; the higher the score, the more pronounced the necessity to take medication and the patient's medication apprehension, representing a stronger belief in taking medication. A previous study confirmed the scale's validity in a population of AR patients receiving SLIT, with Cronbach alpha coefficients of 0.914 and 0.915 for the two dimensions of the scale, and reliability coefficients of 0.901 and 0.819 for the two dimensions of retesting [19].

The self-efficacy scale was developed by Schwarzer [20] and translated into Chinese by Zhang [21]. It is a unidimensional scale with 10 entries; responses are based on a 4-point scale and the summed scores of the 10 entries range from 10 to 40. Higher scores indicate higher self-efficacy. Dong conducted a reliability test among AR patients who underwent SLIT, and the reported total Cronbach alpha coefficient of the scale was 0.930 [18].

The medication knowledge level questionnaire developed by Dong [18] was used to assess participants' medication knowledge; the answers to each item are based on a 4-point scale (i.e., 1-"don't know at all,” 2-"know a little,” 3-"know a lot,” 4-"know very much.”). The scores of the 10 items of the questionnaire are summed to obtain the medication knowledge score; higher total scores signify more medication knowledge among respondents.

### 2.7 Ethics considerations

This study was approved by the Ethics Committee of the First Hospital of Shanxi Medical University (IRB Ref. No.: 2022/K192). Participants were provided with detailed information about the study, and written informed consent was obtained from the participants prior to data collection.

### 2.8 Data collection and statistical analysis

Data were collected via WeChat using Questionnaire Star (a questionnaire tool) at baseline (T0), post-intervention (T1), at 1-month follow-up (T2), and at 3-month follow-up (T3) from participants included in the study.

Data were analyzed using IBM SPSS Statistics for Windows, version 22.0 (IBM SPSS Data Collection, New York, NY, USA). Data on the demographic and clinical characteristics of the participants in the two groups were compared using Student's t -test, Mann–Whitney *U* test, chi-square, or Fisher's exact test, as appropriate. Dropout cases leading to missing data of six patients (three each from the experimental and control groups) were excluded from the analysis. A two-way repeated-measures analysis of variance (ANOVA) was used to explore between-group (group: experimental vs. control), within-group (time: baseline, post-intervention, and two follow-ups), and interaction (group ∗ time) effects. The criterion for statistical significance was set at p < 0.05 in a two-tailed test. An independent-samples *t*-test was used for pairwise comparisons between the two groups, and a paired-samples *t*-test was used to compare outcomes at different time points within the group.

## 3 Results

### 3.1 Participant characteristics

A total of 120 participants who met the inclusion criteria engaged in this study. During the intervention process, one patient in the experimental group stopped treatment due to asthma-related complications, and one patient could not be contacted by phone or WeChat and did not visit the outpatient clinic in time.

Two independent samples t-tests were conducted to analyze measurement data (age, duration of illness, VAS, duration of treatment, etc.); the chi-square test was used to analyze count data (sex, occupational status, marital status, smoking status, treatment， sensitization data, comorbidities, etc.); and the rank sum test for non-parametric statistics was used to analyze rank data (education level, affordable medical expenses, impact of nasal symptoms, impact of place of residence, conscious treatment effect, etc.). The results are presented in Table 2. The general data of the two groups of patients in this study were balanced and comparable.

**Table 2:** Comparison of the results of general participant information between the two groups.

Variable		Experimental（n = 58）	Control（n = 58）	*T*/*Z*/*χ*^*2*^	*P*
Sex	Male	30	29	*χ*^*2*^ = 0.034	0.853^b^
	Female	28	29		
Age		33.38 ± 9.63	31.09 ± 8.84	*t* = 1.336	0.184^a^
Level of education	Primary school	1	2	*Z* = −0.795	0.427^c^
	Junior middle school	2	3		
	Polytechnic school	7	9		
	Senior middle school	14	13		
	Junior college	21	20		
	Bachelor's degree or above	13	11		
Employment	Employed	48	45	*χ*^*2*^ = 2.534	0.631^b^
	Unemployed	10	13		
Affordable medical costs	≤100	0	0	*Z* = −0.685	0.493^c^
	＞ 100 ≤ 200	1	0		
	＞ 200 ≤ 300	37	35		
	＞ 300	20	23		
Marital status	Unmarried	27	24	*χ*^*2*^ = 1.437	0.697^b^
	Married	25	28		
	Divorce	6	5		
	Widowed	0	1		
Smoking status	No smoking	28	26	*χ*^*2*^ = 0.139	0.710^b^
	Smoking	30	32		
Severity (VAS)		6.21 ± 1.95	6.34 ± 1.91	*t* = 1.336	0.810^a^
Treatment	Use antiallergic medicine	47	43	*χ*^*2*^ = 0.793	0.373^b^
	SLIT only	11	15		
Sensitization data	Single dust mite allergy	30	27	*χ*^*2*^ = 0.310	0.577^b^
	Non-monoallergy	28	31		
Time of illness (months)		47.93 ± 24.86	46.86 ± 22.15	*t* = 0.244	0.807^a^
Treatment duration (months)		2.53 ± 2.96	3.17 ± 2.75	*t* = −1.201	0.232^a^
Complication	Allergic conjunctivitis	20	22	*χ*^*2*^ = 1.618	0.951^b^
	Food allergy	17	16		
	Asthma	8	9		
	Eczema	6	5		
	Nettle-rash	4	3		
	Other__	3	3		
Whether nasal symptoms affects treatment	Does not affect	4	5	*Z* = −0.341	0.773^c^
	Does not affect too much	7	8		
	General	21	19		
	Affects to some extent	9	11		
	Affects highly	17	15		
Whether residence affects treatment	Does not affect	5	9	*Z* = −0.607	0.544^c^
	Does not affect too much	13	11		
	General	24	22		
	Affects to some extent	9	10		
	Affects highly	7	6		
Treatment effect	Not good	2	3	*Z* = −0.234	0.815^c^
	General	39	36		
	Good	17	19		

a Independent-samples *t*-test.

b χ2 test.

c Mann-Whitney *U* test.

### 3.2 Intervention effects on outcomes

**Fig. 1:**
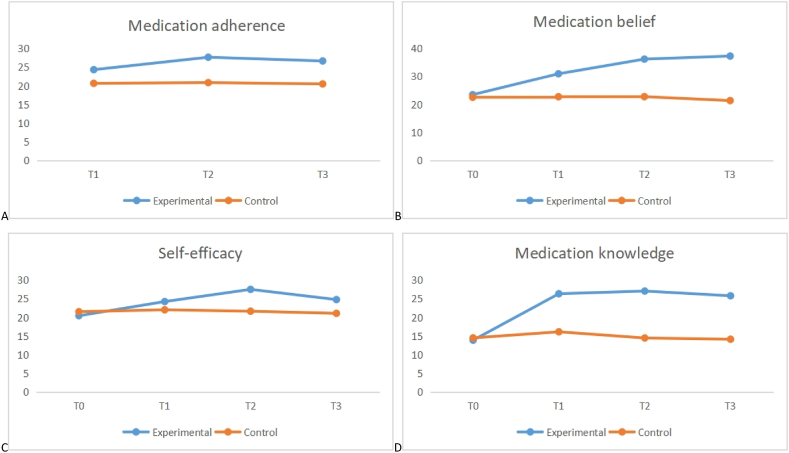
Changes in the mean scores of medication adherence (A), medication beliefs (B), self-efficacy (C), and medication knowledge (D).

Results based on patients’ outcome variables at baseline, post-intervention, and at the two follow-ups are presented in Table 3. Fig. 1(A–D) shows the changes in the mean scores of medication adherence, medication beliefs, self-efficacy, and medication knowledge over time. At T0, no significant differences were noted between the experimental and control groups in medication adherence beliefs (t = 0.88, p = 0.381), self-efficacy (t = −1.37, p = 0.175), or level of medication knowledge (t = −1.07, p = 0.286). The ANOVA results showed significant differences between the experimental and control groups in medication adherence (F = 426.11, p < 0.001), medication beliefs (F = 216.77, p < 0.001), self-efficacy (F = 17.10, p < 0.001), and medication knowledge (F = 483.32, p < 0.001). Further, significant differences over time were observed in medication adherence (F = 51.46, p < 0.001), medication beliefs (F = 216.77, p < 0.001), self-efficacy (F = 88.99, p < 0.001), and medication knowledge (F = 496.04, p < 0.001). Interactions had a significant effect on medication adherence (F = 88.96, p = 0.034), medication beliefs (F = 223.00, p < 0.001), self-efficacy (F = 85.47, p < 0.001), and medication knowledge (F = 273.13, p < 0.001), suggesting that the duration and mode of intervention had an effect on these outcomes for the experimental group participants. A *t*-test of two independent samples was also conducted on medication adherence scores for both groups. The post-intervention scores were significantly higher in the experimental group at T1, T2, and T3 than in the control group during the same periods.

**Table 3:** Baseline comparison of patient outcomes（‾x ± s）.

	Variable	Group	T0 (Mean ± SD)	T1 (Mean ± SD)	T2 (Mean ± SD)	T3 (Mean ± SD)	Between groups F(p)	Within group F(p)	Interaction effect F(p)
**ANOVA analysis**^a^	Medication adherence (range of scores:7−35)	Experimental	/	24.38 ± 3.78	27.72 ± 3.40	26.72 ± 2.96	426.11	51.46	88.96
		Control	/	21.07 ± 4.26	20.88 ± 3.61	20.53 ± 3.43	<0.001	<0.001	<0.001
	Medication beliefs (range of scores:10−50)	Experimental	23.53 ± 5.60	31.03 ± 4.47	36.29 ± 4.04	37.38 ± 3.26	216.77	216.77	223.00
		Control	22.59 ± 6.02	22.79 ± 4.79	22.81 ± 3.86	21.41 ± 3.27	<0.001	<0.001	<0.001
	Self-efficacy (range of scores:10−40)	Experimental	20.47 ± 4.18	24.28 ± 4.08	27.55 ± 3.21	24.81 ± 2.16	17.10	88.99	85.47
		Control	21.57 ± 4.51	22.07 ± 4.11	21.69 ± 4.32	21.12 ± 4.19	<0.001	<0.001	<0.001
	Medication knowledge (range of scores:10−40)	Experimental	13.91 ± 2.83	26.38 ± 2.80	27.10 ± 2.70	25.83 ± 2.41	483.32	496.04	273.13
		Control	14.53 ± 3.22	16.16 ± 2.14	14.50 ± 1.81	14.17 ± 1.77	<0.001	<0.001	<0.001
**Comparison between groups**^b^	**Variable**	**Group**	T0（t, p）	T1（t, p）	T2（t, p）	T3（t, p）			
	Medication adherence	Experimental	/	4.956, <0.001	10.512, <0.001	12.142, <0.001			
		Control							
	Medication beliefs	Experimental	0.879, 0.381	9.582, <0.001	18.391, <0.001	26.328, <0.001			
		Control							
	Self-efficacy	Experimental	−1.366, 0.175	2.901, 0.004	8.296, <0.001	5.965, <0.001			
		Control							
	Medication knowledge	Experimental	−1.071, 0.286	22.074, <0.001	24.851, <0.001	24.569, <0.001			
		Control							

SD Standard deviation.

T0: Before intervention; T1: Two weeks after intervention; T2: First month after intervention; T3: Third month after intervention.

a The outcomes were based on two-way analysis of variance (ANOVA) of repeated measures.

b The outcomes were analyzed using an independent-samples *t*-test.

### 3.3 Satisfaction and feedback

The study showed that the majority of patients (91.4 %) provided positive evaluations (very satis-factory and relatively satisfactory) of the intervention based on the IMB model. No patients were unsatisfied with the intervention. The majority of patients (93.1 %) stated that no questions disturbed them during the interview process. Moreover, the vast majority of patients (96.6 %) were willing to recommend the intervention based on the IMB model to other patient.

## 4 Discussion

The international recommendation for SLIT is 3–5 years and symptom relief begins only 3–6 months after treatment. Patients are required to have good adherence to ensure the treatment effect. Therefore, effective supervision and management of patients by medical staff is required to intervene in long-term compliance. The IMB model is one of the theoretical models for promoting behavioral change. We applied this theoretical framework to the present study to directly or indirectly influence patients' medication adherence by increasing medication knowledge, medication beliefs, and self-efficacy.

Medication adherence scores were generally lower in both groups before the intervention, in line with the results of other studies [[22], [23], [24]]. Medication adherence scores were significantly higher in the trial group than in the pre-intervention group at all three post-intervention time points, and significantly higher than those in the control group during the same period (p < 0.05). these results suggest that an intervention based on the IMB model can be effective in improving medication adherence in SLIT in AR patients. The results may be attributable to the IMB model's emphasis on the important role of motivation and self-efficacy during behavior change and behavior maintenance practices [25]. Studies have indicated [22,26] that medication-related knowledge, self-efficacy, and beliefs about taking medication are important factors influencing medication adherence in SLIT. In this study, the intervention was implemented focusing on the three dimensions of information, motivation, and behavioral skills. In addition to traditional education, the experimental group used the convenience of the WeChat public platform to share articles to improve patients' medication knowledge, motivational interviewing to motivate patients to take medication and enhance their beliefs about taking medication, and behavioral skills such as providing medication record cards and WeChat punch cards to improve patients' self-efficacy. WeChat groups were also used to enhance patients' indirect experience of medication use and reduce the incidence of behaviors such as under-dosing, forgetting about, and discontinuing medication. In our study, the interventions for patients in the control group focused on providing education and answering questions about medication, which had some effect on improving patients' medication knowledge, medication beliefs, and self-efficacy, but the improvement was small. Indeed, medication beliefs and self-efficacy are important factors influencing medication adherence. Our findings suggest that IMB model-based interventions improve medication adherence in AR patients with SLIT in several ways, providing a new approach to clinical medication care.

Medication beliefs refer to patients' perceptions of the usefulness of the medication they are using and their concerns about the possibility of some adverse effects [27,28]. Among them, patients' concerns about the possibility of some adverse reactions to their medication and adverse emotions experienced during medication use are the main factors that could be addressed in interventions [29]. Both groups scored moderately low before the intervention, and after the intervention, the patients' medication belief scores significantly improved in the experimental group. This indicates that the IMB model-based intervention is effective in improving medication beliefs in AR patients. The IMB model-based intervention involved providing patients with adequate information about the disease and medication, alleviating patients' concerns about adverse effects and uncertainty about efficacy, exploring the causes of patients' negative emotions during the motivational interview, and providing guidance to enhance patients' positive attitudes toward taking medication and knowledge about practices to maintain health. In addition, patients appeared to have a more objective perception of treatment during motivational interviewing, which may have encouraged them to address any internal ambivalence regarding medication and guided them to weigh the pros and cons of medication, thereby making them aware of the importance of adherence to medication, increasing their confidence in treating the disease and adhering to medication, stimulating their subjective initiative to take medication, and strengthening their belief in taking medication [30,31].

Self-efficacy refers to whether or/and to what extent an individual believes that they are competent enough to perform a specific task [32]. Self-efficacy is one of the important influencing factors for medication adherence in patients with chronic diseases [33]. Self-efficacy scores were generally lower in both groups before the intervention and higher in the experimental group at all three post-intervention time points than at baseline. The self-efficacy scores of the experimental group tended to decrease after 1 month of the intervention, which may be related to the fact that the other two factors affecting self-efficacy (i.e., medication knowledge and medication beliefs) both tended to decrease after 1 month, but were still significantly different from the scores before the intervention. This suggests that the intervention based on the IMB model can effectively improve self-efficacy pertaining to SLIT in AR patients. Compared with traditional health education, the IMB model-based intervention is a systematic, individualized, and continuous intervention model. Through this interventional approach that considers the dimensions of information, motivation, and behavioral skills, patients have a more objective perception of treatment, and by weighing the pros and cons, they have an in-depth understanding of the possible barriers to overcome in treatment, which further increases their confidence in adhering to their medication. Studies have shown that improving patients' knowledge of their illness can further enhance their self-efficacy with medication; in addition, an increase in patients' beliefs about taking medication can improve self-efficacy to a greater extent [34,35].

The medication-related knowledge scores of patients in both groups were low before the intervention and improved in both groups after the intervention, with a higher increase in patients in the experimental group than in the control group. The medication-related knowledge scores showed a decreasing trend 1 month after the intervention, probably because the patients thought they had acquired enough information and paid less attention to it, or may have forgotten some of it; however, there was no significant difference from the post-intervention time point. This suggests that an intervention based on the IMB model can be effective in improving medication knowledge in AR patients. The IMB model-based intervention, in terms of the steps taken in the information dimension, involved sharing an information booklet on medication knowledge with the patients as well as educational articles on the WeChat platform, and establishing a WeChat group in which healthcare professionals provided relevant knowledge and answered questions to establish good interactions with the patients and understand their level of knowledge on medication, so that targeted guidance could be provided to them. SLIT is a long-term process, and its health education needs to be repeated, continuous and continuously updated [16]. The WeChat public will also be constantly updated with health education knowledge after the study is completed. In the WeChat group, patients not only obtained health information from healthcare professionals but also communicated with other patients to gain indirect knowledge on other patients’ experiences, which helped improve their medication adherence.

## 5 Conclusions

This study used the IMB model as a theoretical framework to develop an intervention to improve medication adherence in patients with AR receiving SLIT. Our results confirmed that the intervention based on the IMB model was effective in improving medication adherence, beliefs regarding taking medication, self-efficacy, and medication knowledge in patients with AR undergoing SLIT. Based on the IMB model, which constitutes the theoretical framework of this study, we propose that an increase in medication adherence may be achieved through an increase in patients' medication knowledge, medication beliefs, and self-efficacy. Therefore, an intervention based on the IMB model is an effective way to improve medication adherence in AR patients receiving SLIT and may provide a reference for medication care in outpatient settings.

## 6 Limitations

Despite its important findings, this study has some limitations that must be acknowledged. First, due to time constraints, the follow-up period was only 3 months, which is a short follow-up period to monitor ongoing intervention effects on patients' medication adherence. It is recommended that the follow-up period be appropriately extended in future studies to observe the long-term effects. Second, the intervention protocol in this study required the use of a WeChat platform, which requires patients to have a smartphone and be able to use the WeChat function, which to some extent may have limited the inclusion of older patients, less educated patients, and patients who are not skilled in using smartphones. The population included in this study was relatively limited; we recommend that future studies develop targeted interventions for patients who do not have access to smartphones. Finally, the outcome indicators in this study did not include the quality of life and symptom scores of patients; future studies could consider their inclusion. Finally, the outcome indicators of this study did not include patients' symptom scores or quality of life; we will next conduct a three-year follow-up to assess the impact of the intervention on clinical symptoms and quality of life.

## Funding

Project of Shanxi Science and Technology Association (JKKP202123).

## Data availability statement

Data included in article/supp. material/referenced in article.

## Additional information

No additional information is available for this paper.

## CRediT authorship contribution statement

**Qian Wang:** Conceptualization, Data curation, Investigation, Methodology, Writing - original draft. **Ruifang Zhu:** Conceptualization, Formal analysis, Resources, Validation, Visualization, Writing - review & editing. **Yingzi Cao:** Investigation, Methodology, Software, Validation, Visualization. **Yan Ning:** Investigation, Methodology, Software, Supervision. **Yaoqing Feng:** Data curation, Investigation. **Yan Feng:** Funding acquisition, Project administration, Supervision, Visualization. **Shifan Han:** Conceptualization, Data curation, Formal analysis, Funding acquisition, Supervision.

## Declaration of competing interest

The authors declare that they have no known competing financial interests or personal relationships that could have appeared to influence the work reported in this paper.

## References

[1] Bousquet J. (2020). Allergic rhinitis. Nat. Rev. Dis. Prim..

[2] Subspecialty Group of Rhinology (2022). Chinese Medical Association, [Chinese guideline for diagnosis and treatment of allergic rhinitis (2022, revision)]. Zhonghua er bi yan hou tou jing wai ke za zhi.

[3] Dykewicz M.S. (2020). Rhinitis 2020: a practice parameter update. J. Allergy Clin. Immunol..

[4] Zhang Y., Lan F., Zhang L. (2021). Advances and highlights in allergic rhinitis. Allergy.

[5] Zhang Y., Lan F., Zhang L. (2022). Update on pathomechanisms and treatments in allergic rhinitis. Allergy.

[6] Field K., Blaiss M.S. (2020). Sublingual versus subcutaneous immunotherapy for allergic rhinitis: what are the important therapeutic and real-world considerations?. Curr. Allergy Asthma Rep..

[7] Wise S.K., Schlosser R.J. (2012). Subcutaneous and sublingual immunotherapy for allergic rhinitis: what is the evidence?. Am J Rhinol Allergy.

[8] Liu W. (2021). Compliance, efficacy, and safety of subcutaneous and sublingual immunotherapy in children with allergic rhinitis. Pediatr. Allergy Immunol..

[9] Hamine S. (2015). Impact of mHealth chronic disease management on treatment adherence and patient outcomes: a systematic review. J. Med. Internet Res..

[10] Náfrádi L., Nakamoto K., Schulz P.J. (2017). Is patient empowerment the key to promote adherence? A systematic review of the relationship between self-efficacy, health locus of control and medication adherence. PLoS One.

[11] Minhat H.S., Zakaria L.N. (2022). Information-Motivation-Behavioural Skills Model-based intervention effectively improved hygiene-related self-efficacy and practice among the primary caregivers of the under-three indigenous children in Malaysia. Zoonoses Public Health.

[12] Tuthill E.L. (2017). Exclusive breast-feeding promotion among HIV-infected women in South Africa: an Information-Motivation-Behavioural Skills model-based pilot intervention.

[13] Lim K.E. (2022). Self-management model based on information-motivation-behavioral skills model in patients with chronic obstructive pulmonary disease. J. Adv. Nurs..

[14] Akbari M. (2022). Psychological predictors of treatment adherence among patients with diabetes (types I and II): modified information-motivation-behavioural skills model. Clin. Psychol. Psychother..

[15] Man Y. (2018). An individualised medication management programme based on the information-motivation-behavioural skills model in patients with Parkinson's disease. Chinese Nursing Management.

[16] Li H. (2019). Chinese guideline on sublingual immunotherapy for allergic rhinitis and asthma. J. Thorac. Dis..

[17] Zhang R. (2015).

[18] Dong Y. (2018).

[19] Yan, F., et al., Evaluation of the applicability of the Chinese version of the modified medication belief scale (BQM) to sublingual immunotherapy in patients with allergic rhinitis, Chin. J. Health Statistics. 36(3) 2019 354-357.

[20] Schwarzer R. (1997). The assessment of optimistic self-beliefs: comparison of the Chinese, Indonesian, Japanese, and Korean versions of the general self-efficacy scale. Psychologia.

[21] Zhang J.X., Schwarzer R. (1995). Measuring optimistic self-beliefs: a Chinese adaptation of the general self-efficacy scale. Psychologia.

[22] Imanaka T. (2019). An analysis of factors associated with compliance and dropout of sublingual immunotherapy on Japanese cedar pollinosis patients. Int Forum Allergy Rhinol.

[23] Malet A. (2016). Comprehensive study of patients' compliance with sublingual immunotherapy in house dust mite perennial allergic rhinitis. Adv. Ther..

[24] Liu Y. (2016). Compliance of sublingual immunotherapy in 161 patients with allergic diseases. Chinese J. Allergy and Clinical Immunol..

[25] Dzerounian J. (2022). Health knowledge and self-efficacy to make health behaviour changes: a survey of older adults living in Ontario social housing. BMC Geriatr..

[26] Cea-Calvo L. (2020). Different associations of intentional and non-intentional non-adherence behaviors with patient experience with healthcare and patient beliefs in medications: a survey of patients with chronic conditions. Patient Prefer. Adherence.

[27] Olorunfemi O., Ojewole F. (2019). Medication belief as correlate of medication adherence among patients with diabetes in Edo State, Nigeria. Nurs Open.

[28] Al-Noumani H. (2019). Health beliefs and medication adherence in patients with hypertension: a systematic review of quantitative studies. Patient Educ. Counsel..

[29] Brown M.T. (2016). Medication adherence: truth and consequences. Am. J. Med. Sci..

[30] u P. (2021). Patients' characterization of medication, emotions, and incongruent perceptions around adherence. J. Personalized Med..

[31] Ertem M.Y., Duman Z. (2019). The effect of motivational interviews on treatment adherence and insight levels of patients with schizophrenia: a randomized controlled study. Psychiatr. Care.

[32] Eller L.S. (2018). Describing self-care self-efficacy: definition, measurement, outcomes, and implications. Int J Nurs Knowl.

[33] Ma Q. (2018). Self-reported reasons for treatment non-adherence in Chinese asthma patients: a 24-week prospective telephone follow-up study. The clinical respiratory journal.

[34] Kizza I.B., Maritz J. (2019). Family caregivers for adult cancer patients: knowledge and self-efficacy for pain management in a resource-limited setting. Support. Care Cancer.

[35] Mckinney C.O. (2016). Racial and ethnic differences in breastfeeding. Pediatrics.

